# An Overview on Radiation Sensitivity in Hereditary Breast and Ovarian Cancer Syndrome

**DOI:** 10.3390/cancers14133254

**Published:** 2022-07-02

**Authors:** Diana Gonçalves, Ana Salomé Pires, Inês A. Marques, Inês Gomes, Gabriela Sousa, Maria Filomena Botelho, Ana Margarida Abrantes

**Affiliations:** 1 Coimbra Institute for Clinical and Biomedical Research (iCBR) Area of Environment, Genetics and Oncobiology (CIMAGO), Institute of Biophysics, Faculty of Medicine, University of Coimbra, Azinhaga de Santa Comba, 3000-548 Coimbra, Portugal; dianapgoncalves.1998@gmail.com (D.G.); pireslourenco@uc.pt (A.S.P.); ines.marques@student.uc.pt (I.A.M.); inescf_gomes@hotmail.com (I.G.); mfbotelho@fmed.uc.pt (M.F.B.); 2 Faculty of Science and Technology, University of Coimbra, Rua Sílvio Lima, 3030-790 Coimbra, Portugal; 3 Center for Innovative Biomedicine and Biotechnology (CIBB), University of Coimbra, Azinhaga de Santa Comba, 3000-548 Coimbra, Portugal; 4 Clinical Academic Center of Coimbra (CACC), Azinhaga de Santa Comba, 3000-548 Coimbra, Portugal; 5 Faculty of Pharmacy, University of Coimbra, Azinhaga de Santa Comba, 3000-548 Coimbra, Portugal; 6 Portuguese Oncology Institute of Coimbra Francisco Gentil, Avenida Dr. Bissaya Barreto nº98, 3000-075 Coimbra, Portugal; gsousa2140@gmail.com; 7 Medical Imaging and Radiotherapy Department, Polytechnic Institute of Coimbra, ESTESC-Coimbra Health School, Rua 5 de Outubro, 3046-854 Coimbra, Portugal

**Keywords:** hereditary breast and ovarian cancer, *BRCA1*, *BRCA2*, ionizing radiation

## Abstract

**Simple Summary:**

Individuals with hereditary breast and ovarian cancer (HBOC) syndrome are more likely to develop several types of cancer compared to the general population. They are regularly subjected to diagnostic exams and therapeutic options that use ionizing radiation, making it important to understand the effects that this can induce. Thus, several studies have been carried out to understand whether the exposure of individuals with HBOC to ionizing radiation may be associated with the onset of cancer. However, the results are inconsistent.

**Abstract:**

Hereditary breast and ovarian cancer (HBOC) syndrome is a condition in which individuals have an increased risk of developing different types of cancer when compared to the general population. *BRCA1* repair associated (*BRCA1*) and *BRCA2* repair associated (*BRCA2*) genes are tumor suppressor genes that play a crucial role in cell, by repairing DNA damage. Mutations in these genes are responsible for 25% of HBOC cases. Individuals with this syndrome are often subjected to diagnostic imaging techniques, as well as therapeutic options, that use ionizing radiation, so it is crucial to understand whether these individuals may present higher radiosensitivity and, therefore, its consequences. Several studies have been carried out to understand if the exposure to different ionizing radiation doses can induce cancer in individuals with HBOC. Some of these studies have shown that individuals with HBOC are hypersensitive to the ionizing radiation and, therefore, have neoplasms resulting from mutations in genes that are important in maintaining genomic stability. When mutated, genes no longer guarantee this stability and promote the induction of carcinogenesis. Oppositely, other studies show that there is no association between exposure to ionizing radiation and an increased risk of developing cancer. Thus, the results are inconsistent, and there is a great need to clarify this relationship. In this review, we present the characteristics of HBOC syndrome and the effects that ionizing radiation can induce in individuals who have it. In addition, we review the studies that have already been carried out on this subject.

## 1. Hereditary Breast and Ovarian Cancer Syndrome

Cancer is a heterogeneous and mostly non-hereditary disease. Considering breast and ovarian cancers, most occur sporadically, resulting from mutations that occur in somatic cells. However, there are cases where genetic alterations are transmitted to offspring. Hereditary breast and ovarian cancer (HBOC) is an autosomal dominant inherited syndrome characterized by a high risk of breast cancer, in both genders, associated or not with ovarian cancer, including fallopian tube cancer and primary peritoneal cancer, in females [1,2].

HBOC is mostly caused by germline deleterious mutations in *BRCA1* DNA repair associated (*BRCA1*) and *BRCA2* DNA repair associated (*BRCA2)* genes and they affect all ethnic groups and races. In the general population, excluding the Ashkenazi Jewish population, mutations in *BRCA1* and *BRCA2* genes are estimated to have a frequency between 1:400 and 1:500. However, in the Ashkenazi Jewish population, the frequency of causal variants is 1 in 40 [1,2]. These genes are tumor suppressor genes that play a crucial role in the cell, so when mutated they lead to the development of dysfunctional proteins, unable to exert the DNA repair function. Non-repaired DNA leads to genetic instability with an increased risk of malignancy [2,3]. Affected individuals tend to develop these types of cancer earlier, usually before 50 years of age [1,2,4].

In women who do not carry the mutations in *BRCA* genes, the lifetime risk of developing breast cancer or ovarian cancer is approximately 12% or 1–2%, respectively. However, when there are mutations in the *BRCA1* and *BRCA2* genes, the risk of breast cancer increases considerably to 46–87% for mutations in the *BRCA1* gene and 38–84% for mutations in the *BRCA2* gene; in addition, the risk of ovarian cancer is 39–63% for mutations in the *BRCA1* gene and 15.5–27% for mutations in the *BRCA2* gene. Regarding men with mutations in the *BRCA2* gene, as well as *BRCA1* mutations (although with lower associated risks), they also have a higher risk of developing cancer, namely breast cancer and prostate cancer. Less frequently, those mutations may also be associated with an increased risk of developing other cancers, such as pancreatic cancer and melanoma. However, melanoma seems to only be associated with individuals carrying mutations in the *BRCA2* gene [1].

Although mutations in the *BRCA1* and *BRCA2* genes are mainly responsible for HBOC, only 25% of cases are associated with them. The exome sequencing technique allowed the discovery of substantial locus heterogeneity among affected families that do not carry mutations in *BRCA1* or *BRCA2,* showing that other genes are associated with HBOC. Currently, more than 25 genes have been identified to be associated with this syndrome, including the checkpoint kinase 2 (*CHEK2*) gene, ATM serine/threonine kinase (*ATM*) gene and partner and localizer of the *BRCA2* (*PALB2*) gene. Most of these genes encode proteins that act on genome maintenance pathways in conjunction with the *BRCA1* and *BRCA2* genes [5].

According to the last update in 2022 by Petrucelli et al., *BRCA1*- and *BRCA2*-associated HBOC should be suspected when an individual presents a personal history or family history with a first-, second- or third-degree relative who exhibits one of the following features: (1) ovarian cancer; (2) breast cancer diagnosed at an age below 50 years old; (3) male breast cancer; (4) several primary breast cancers in one or both breasts; (5) triple-negative breast cancer, particularly when diagnosed before age 60; (6) a combination of prostate cancer and/or pancreatic cancer with breast cancer and/or ovarian cancer; (7) breast cancer diagnosed at any age in an individual of Ashkenazi Jewish descent; (8) two or more relatives, one under 50 years of age, with breast cancer; (9) three or more relatives at any age with breast cancer; or (10) previous identification of a *BRCA1* or *BRCA2* pathogenic variant in the family [1].

In individuals who do not meet the above criteria, several predictive mathematical models can be used to estimate the probability of having a variant in one of the two genes (*BRCA1* or *BRCA2*); if a result is above a certain threshold, usually 10%, genetic testing should be performed [1]. Among these models, the BRCAPRO statistical model stands out. This model is a risk assessment tool associated with the identification of pathogenic mutations in the *BRCA1* and *BRCA2* genes using a Mendelian approach. This software incorporates patients’ personal and family history, including the number of tumors of the patients, as well as of their first-, second-, and third-degree relatives [6]. However, this model has some disadvantages because it does not incorporate disease characteristics, for example triple-negative breast cancer. Thus, the Myriad model and the Manchester score are also used [7].

Regarding the diagnosis of HBOC associated with mutations in *BRCA1* and *BRCA2* genes, which are linked to most families who have the syndrome, it is established through the identification of a heterozygous germline pathogenic variant in these genes, through molecular genetic tests. The ideal candidate to take these tests must have at least one of the characteristics previously mentioned and, only after, must it be carried out on family members. If it is not possible to perform the molecular test on individuals with a personal history of cancer, then the test can be performed on a healthy family member. However, it is necessary to emphasize that a negative result does not exclude the possibility that there is a pathogenic variant in the *BRCA1* or *BRCA2* genes in the family [1].

Genetic tests identify changes in chromosomes, genes, or proteins, and can be divided into molecular, cytogenetic, and biochemical tests. Molecular genetic tests, used in this syndrome, assess one or more genes to identify variations or mutations responsible for genetic diseases [8]. When no genetic alteration is identified, it is important to make it clear to the patient that the risk of developing cancer is the same as that of the general population. However, if the test identifies a causal variant, it is crucial to test family members for that specific alteration to provide adequate follow-up [6]. Early diagnosis in carriers of pathogenic variants of the *BRCA1* and *BRCA2* genes is important for the application of effective surveillance and prophylactic measures. The goal is the prevention or, when this is not possible, the early diagnosis of malignant neoplasms. Thus, there is surveillance, through imaging tests, some of which use ionizing radiation, and prevention programs suited to the type of mutation identified. Regarding prevention, risk-reducing mastectomy and chemoprevention have been used for breast cancer, and risk-reducing oophorectomy for ovarian cancer [1].

### BRCA1 and BRCA2 Genes

DNA, which harbors genetic information, is the most important structure at the cellular level and is constantly suffering damage caused by internal and external factors. Repairing these injuries is crucial for the proper cell function [2]. Tumor suppressor genes are important for the normal functioning and development of the organism, constituting the key to the regulation of cell division. From these genes, specific proteins are encoded with several functions in the cell [9].

The *BRCA1* and *BRCA2* genes were first linked to breast and ovarian cancer susceptibility by Mick and colleagues in 1994 (*BRCA1*) and by Wooster et al., in 1995 (*BRCA2*) [10].

**Figure 1:**
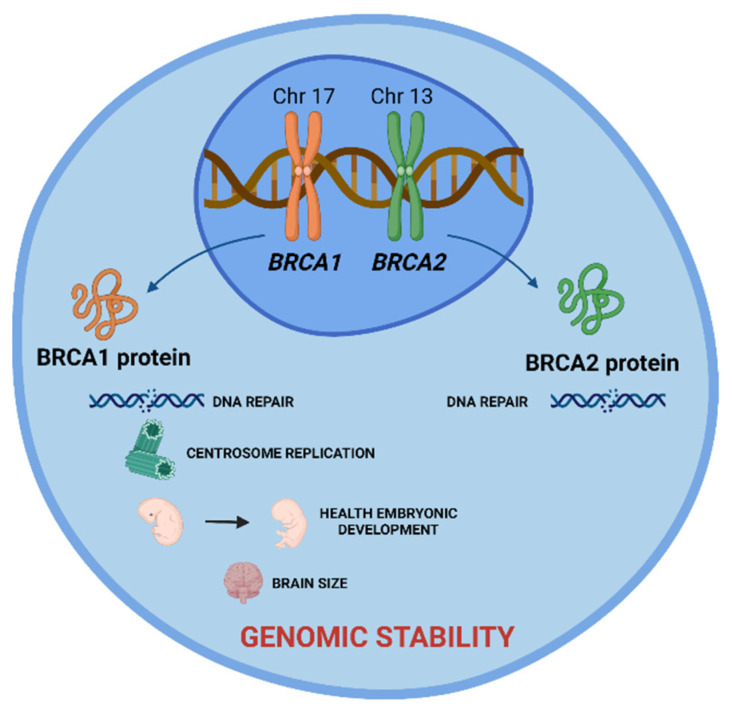
*BRCA1* and *BRCA2* tumor suppressor genes and the role of their proteins in cell. BRCA 1 and BRCA2 proteins are encoded by these genes and play a crucial role in DNA damage repair, allowing the maintenance of genomic stability. Additionally, BRCA1 protein is involved in centrosome replication, health embryonic development, and brain size. Created with BioRender.com.

These are tumor suppressor genes responsible for repairing DNA double-strand breaks, allowing genomic stability to be maintained. In addition to the repair function, these genes control centrosome dynamics, chromosomal segregation, and cytokinesis, and stabilize the genome temporally and spatially in the cell cycle. Thus, an interruption in these cellular functions leads to genomic instability as well as the emergence of a carcinogenic environment. In addition to the different roles that these genes play, *BRCA1* is involved in other functions, such as in onset of breast and ovarian cancer, healthy embryonic development, centrosome replication, and brain size (Figure 1) [2].

The *BRCA1* gene is located in region 21.31 of the long arm of chromosome 17 (17q21.31). It is composed of 24 exons, 22 of which are coding, spread over 100 kb of genomic DNA. More than 1700 mutations in this gene have been described, with more than 800 being associated with tumor incidence susceptibility. This gene is responsible for encoding and synthesizing the BRCA1 protein (Figure 1) [10,11,12].

**Figure 2:**
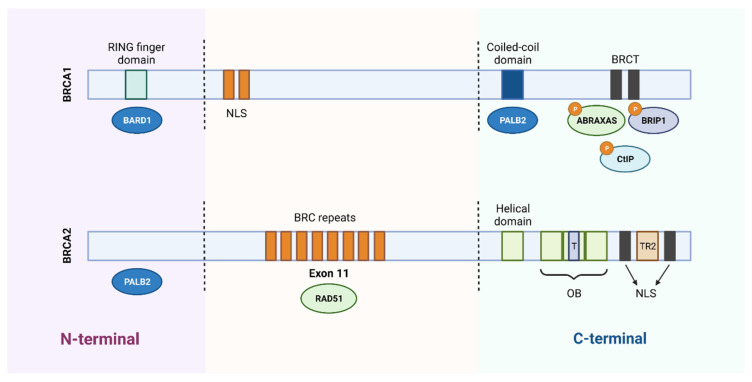
Schematic representation of the functional domains of BRCA1 and BRCA2 proteins. BRCA1 protein consists of 1863 amino acids and BRCA2 protein of 3418 amino acids. The N-terminal region of BRCA1 contains a RING domain that associates with the BARD1 protein. The central region of the protein contains two nuclear localization signals (NLS) and the C-terminal of BRCA1 contains a coiled-coil domain that associates with PALB2 and two BRCT domains that mediate interaction with different proteins. Regarding BRCA2, it binds to PALB2 through the N-terminal region and contains 8 BRC repeats, in the central region, responsible for its association with the RAD51 protein. In the C-terminal region, it contains a DNA-binding domain, consisting of a helical domain, a tower domain (T) and three oligonucleotide binding (OB) domains, which facilitates the binding of BRCA2 to DNA lesions. Furthermore, the carboxy terminal contains two NLS domains and a TR2 domain. Created with BioRender.com.

The BRCA1 protein consists of three essential domains for its multiple functions: an amino terminal region (N-terminal), a central region, and a carboxyl terminal (C-terminal). The N-terminal region contains the RING finger domain, which is essential for the association with the BRCA1-associated RING domain protein 1 (BARD1) and formation of the BRCA1 complex with the E3 ubiquitin ligase. The central part of BRCA1 contains two nuclear localization signals (NLS) and a CHEK2 phosphorylation site. Finally, the C-terminal is constituted by the coiled-coil domain, which associates with PALB2, and by two BRCA1 C-terminal (BRCT) domains that mediate the interaction with important proteins, such as BRCA1-A complex subunit (ABRAXAS), BRCA1-interacting protein C-terminal helicase 1 (BRIP1) and C-terminal binding protein 1 interacting protein (CtIP). Several inherited cancer-associated BRCA1 mutations have been found within the RING domain and BRCT domains, which indicates that both domains are involved in the suppression of breast and ovarian cancer (Figure 2) [10,13].

The *BRCA2* gene is located on the long arm of chromosome 13 in region 12.3 (13q12.3). It is composed of 27 exons, of which 26 are coding, distributed over 384 kDa. More than 1800 mutations associated with this gene are described [10,14,15].

Like the BRCA1 protein, the BRCA2 protein is made up of two terminals, the N-terminal and the C-terminal, and a central region. The N-terminal region contains the transcriptional activation domain (TAD) and binds PALB2. The central region of the BRCA2 protein is encoded by exon 11 and contains 8 BRC repeats that bind to RAD51. At the C-terminal, the DNA-binding domain is located, which contains a helical domain, a tower domain and three oligonucleotide binding (OB) domains that facilitate the binding of BRCA2 to both single- and double-stranded DNA damage. This terminal also contains two NLS domains and a TR2 domain (Figure 2) [10,13].

## 2. Ionizing Radiation

Radiation is energy in the form of electromagnetic waves or particles, emitted from a source. The transmission of energy can take place through a material, such as tissues, or even in space (vacuum) [16]. Radiation can be classified into two main categories regarding the consequence of the interaction with matter: non-ionizing and ionizing radiations. Ionizing radiation is defined as radiation that has enough energy to ionize atoms or molecules by ejecting electrons from them, unlike non-ionizing radiation, which does not have this ability. The ejected electron may have sufficient kinetic energy to produce other ionization as it passes through matter. Ionizing radiation can be electromagnetic radiation, as X-rays and γ-rays, or particulate radiation, as α particles, β particles, neutrons, or protons [17,18].

Several medical applications use ionizing radiation, contributing to an increased exposure of individuals to it. Most of this exposure comes from nuclear medicine diagnostic tools that use gamma radiation, but also from radiology through the use of CT scans and standard X-ray technology to diagnose diseases and injuries. Ionizing radiation can also be used for treatment purposes, in radiotherapy and nuclear medicine fields [19].

When crossing the human tissue, ionizing radiation can interact with it by providing energy, which can lead to biological changes. The absorption of the energy deposited by this type of radiation can result in catastrophic damage at the cell, especially in the DNA, in the most radiosensitive structure. However, although ionizing radiation has effects on the tissues and organs it passes through, and the benefits associated with it, such as diagnosis and therapy, outweigh the risks [19].

For a given medium, the rate of energy loss depends not only on the type of radiation and its energy, but also on the density of the irradiated material. Thus, the linear energy transfer (LET) is one of the important factors influencing the biological effect of radiation. In general, the higher the LET, the greater the biological effect. Moreover, charged particles, such as the alpha particles, in general, have a higher LET than X-rays and γ-rays [20,21]. There are two other factors that affect the cellular response to ionizing radiation: the relative biological effectiveness (RBE) and the oxygen enhancement ratio (OER). The RBE is used to quantify biological damage produced by a type of radiation, and it describes the relative effect of LET. This factor increases with increasing LET up to a maximum value. Above this value, the increase in LET does not contribute to more cell damage [20,21,22]. The OER describes the effect of oxygen on the radiation sensitivity of cells. Effectively, the biological effects of radiation are greater when in an aerobic situation, since, in these conditions, higher levels of free radicals are created during the ionization of water. For low-LET radiation, the presence of oxygen in the cell medium dramatically affects the effect of the radiation [23]. Concerning the biological effects of radiation, these can be classified as direct or indirect effects. Direct effects occur when radiation directly deposits its energy on cellular components, critical to the survival of the cell, such as DNA, messenger RNA, membrane lipids, or enzymes, causing alterations. This interaction can affect the cell’s ability to reproduce and, thus, survive. This type of effect is usually associated with high radiation doses and high-LET radiation, as α particles and neutrons [21,24]. On the other hand, indirect effects are associated with the radiolysis of water, the main constituent of cells, or other organic molecules present in the cell, causing the production of radical and non-radical species. When radiation interacts with a water molecule, a sequence of reactions occurs leading to the formation of reactive species responsible for the indirect effects of radiation. Hydroxyl free radical and hydrogen peroxide are responsible for approximately two-thirds of all damage from water radiolysis. Free radicals have unpaired electrons in their structure, which makes them very reactive and susceptible to interacting with DNA molecules, being capable of inactivating cellular mechanisms and causing alterations in the genetic mechanisms of cells because of damage to DNA, proteins, and/or lipids [25]. In the end, these radicals may cause the impairment of cell function or even death. Thus, as the name indicates, these effects indirectly affect the molecules, requiring an intermediate step to reach them [21,24]. This type of effect is caused mainly by ionizing electromagnetic radiation, X-rays and γ-rays, and low-LET radiation. Furthermore, since water constitutes about 60% of the cellular composition, most radiation-induced damage results from the indirect action of radiation [21,24,26].

Ionizing radiation can damage cell membrane, organelles, and other cell constituents. However, the most radiosensitive region of the cell is the DNA molecule, whose damage can cause genomic instability and cancer. Regarding the radiation-induced damage on the DNA molecule, this can lead to structural damage and interfere with genetic transcription. These lesions may involve changes in the bonds between bases (substitution, addition, or deletion of bases), cross substitution, single strand breaks (SSB), and double strand breaks (DSB) [27].

The SSB involves a break in only one of the DNA strands, being produced essentially by low-LET radiation. Since the nitrogenous bases are complementary, this type of damage is relatively easy to repair, because the opposite strand serves as a template to fill the gap [28]. Therefore, repair of DNA damage begins with damage identification, in which two DNA checkpoints monitor damage at specific points within the cell cycle and stop it, allowing time for damage repair [27]. When an SSB is detected, the repair mechanisms used are the base excision repair (BER), the nucleotide excision repair (NER), or mismatch repair (MMR). In BER, the damaged base is removed by DNA glycosylases and then the missing nucleotide is replaced by a specialized repair polymerase. After placing the nucleotide, the DNA ligase seals the cut in the DNA strand and, finally, the broken phosphodiester bonds are re-established, and the damage is repaired [27]. BER is the main mechanism that handles the spontaneous DNA damage caused by free radicals and other reactive species generated by metabolism. Furthermore, it corresponds to the main repair mechanism for small base changes in DNA, such as those caused by oxidation, alkylation, and deamination [29]. On the other hand, the NER mechanism is used to remove extensive lesions and to correct bases that distort the DNA double helix. The helicase opens the DNA, the modified nucleotides are removed along with a surrounding piece of DNA, the DNA polymerase replaces the missing DNA, and finally, the DNA ligase closes the gap [30,31]. Related to the MMR mechanism, incorrect base–base pairs and small insertion–deletions generated during DNA replication are corrected, ensuring genomic stability [30,32]. This mechanism is available in prokaryotic and eukaryotic cells and comprises several proteins involved in lesion recognition, initiation of lesion repair and excision, and finally, DNA resynthesis. Since MMR reduces the number of errors associated with replication, defects in this pathway increase the spontaneous mutation rate and are associated with carcinogenesis [32].

DSB involve breaks in both DNA strands at the same or nearby locations. These breaks are more likely to occur with high-LET radiation and are more likely to result in a defective repair because the molecule may break apart and the mold to guide the repair may be lost. Most DSBs are lethal because damages are detected during replication by cell cycle checkpoints, not allowing the cell to progress in the cell cycle and complete the division process. Since DSBs can result in the loss of large chromosomal regions, they are considered the type of DNA damage that is more complex to repair [28].

Although DSB are more severe than SSBs, due to the breakage resulting in two free ends of DNA, the cell has mechanisms for repairing DSBs: homologous recombination (HR) and non-homologous end joining (NHEJ). HR corresponds to a process in which repair proteins locate homologous DNA, which has the same relative sequence as the damaged DNA, or the sister chromosome. Then, the damaged DNA is resected, and strand invasion occurs. Resection corresponds to a process by which DNA adjacent to the damage is removed from the 5′ end of the break, while strand invasion occurs at the 3′ end of the break. Finally, the lost DNA is synthesized and, when repair of the damage has been completed, the chromosomes separate. This process occurs during the S and G2 phases of the cell cycle due to the availability of a greater number of sister chromatids. On the other hand, NHEJ acts in the G1 phase of the cell cycle and is referred to as “non-homologous”, since this process does not require a homologous template for repair; the broken ends of DNA are just linked together [21,27]. NHEJ and HR mechanisms compete for a DSB, or the choice can be determined by the structure of the broken ends. NHEJ is faster and more efficient, and HR is more complex. Mammals mainly rely on NHEJ for DSB repair, whereas HR generally dominates in organisms with a small genome [33].

BRCA1 protein plays an important role in DSB repair through HR. It recruits the BRCA2 protein to the site where DNA DSB occurred during HR. Additionally, the BRCA1 protein is also involved in the regulation of the NHEJ repair pathway. The interaction of BRCA1 with a factor involved in this mechanism, Ku80, stabilizes the Ku heterodimer at the sites where the lesion occurred, enabling its precise repair. The BRCA2 protein, such as BRCA1, plays a crucial role in HR through mechanisms involving the recruitment of RAD51 to the site of DNA damage, leading to its repair [10].

## 3. Ionizing Radiation and *BRCA1* and *BRCA2* Mutations

Ionizing radiation is responsible for causing dose-dependent DNA damage and, thus, induces the activation of relevant signaling pathways such as DNA repair, cell cycle control, and cell death. When considering exposure to low doses (<100 mSv), used during diagnostic medical imaging techniques such as mammography and chest x-rays, cells show a certain adaptative response and the damage to critical cell structures is low. The adaptive response, described by Olivieri et al., is defined as the induction of cellular resistance to genotoxic effects caused by subsequent exposure to high-dose radiation. Additionally, damage that occurs at the DNA level can, in general, be repaired, with recovery of function [34,35,36]. For exposure to high doses, used in radiotherapy as an oncological treatment, they exhibit a high potential to induce cellular damage in healthy cells, such as SSBs and DSBs. Since DSBs are very severe injuries, they are difficult to repair and can eventually result in the development of a new cancer [37]. However, although in the literature the effects of radiation seem to be well defined, in individuals with mutations in the *BRCA1* and *BRCA2* genes, there is still a great deal of controversy regarding the effects of low and high doses of radiation.

*BRCA1* and *BRCA2* genes play a key role in repairing DNA damage, which can arise because of ionizing radiation, and in regulating the cell cycle, leading to genomic stability and tumor suppression. Ionizing radiation can cause DNA damage, either directly through ionization or indirectly through radiolysis of water. Changes at the DNA level, such as DNA base changes, cross-linking, and DNA SSBs or DSB, are known to cause cancer.

**Figure 3:**
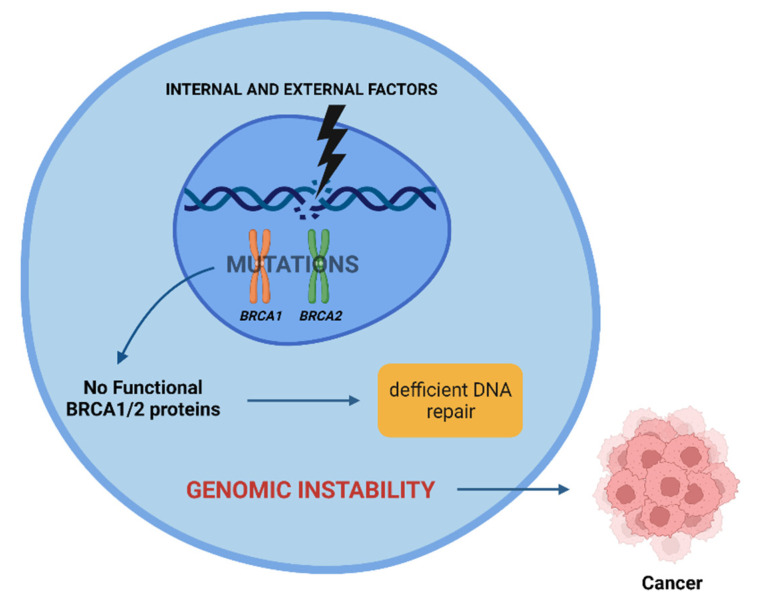
Effect of internal and external factors on *BRCA1* and *BRCA2* genes. Mutations in these genes lead to inefficient DNA repair, contributing to genomic instability. This instability can lead to cancer. Created with BioRender.com.

Radiation-induced carcinogenesis may occur in individuals who have mutations in the *BRCA1* and *BRCA2* genes, as proteins encoded by them are essential in repairing DNA damage. A loss of function in one of the proteins encoded leads to inefficient DNA repair and an increase in genomic instability, sometimes culminating in cancer (Figure 3) [2,38].

This explains the fact that individuals who have mutations in the *BRCA1* and *BRCA2* genes have an increased risk of developing different types of cancer, such as breast and ovarian cancers. Individuals with HBOC are often subjected to diagnostic exams and therapeutic options that use ionizing radiation. For example, women initiate mammography at very early ages, employing a diagnostic tool that uses ionizing radiation. Thus, it is important to understand the role of diagnostic and therapeutic doses in patients with HBOC and whether the capacity to repair DNA damage is different between carriers and non-carriers of mutations in the *BRCA* genes.

### 3.1. Diagnostic Doses

To clarify whether individuals carrying these mutations are more radiosensitive, several retrospective cohort studies have been carried out over the years, in humans, which evaluated the effect of exposure to chest radiographs and mammograms on the development of cancer, namely breast cancer (Table 1) [39].

In 1997, Sharan et al., addressed for the first time the hypothesis that individuals with mutations in the *BRCA2* genes could present greater radiosensitivity when compared to the general population. This study, although inconclusive, was crucial as it raised concerns about the possible consequences of radiation in patients with mutations in the *BRCA2* gene [40]. Later, in 2006, Narold et al., carried out a study that aimed to analyze whether exposure to ionizing radiation from mammography by individuals carrying mutations in the *BRCA1* and *BRCA2* genes was associated with an increased risk of developing breast cancer. This study involved 3200 women previously identified with mutations in these genes, 1600 of whom had been diagnosed with breast cancer. The others (*n* = 1600) were healthy carriers, constituting the control group. Each of the selected individuals answered a questionnaire in which it was possible to obtain information regarding the number of mammograms that each woman had already performed, as well as their age at the time of the first mammogram. The results obtained from this study did not show any association between mammography-related exposure to ionizing radiation and the risk of developing cancer [41].

Also in 2006, Goldfrank et al., carried out another study to investigate whether there was a relationship between the exposure of carriers of mutations in the *BRCA1* and *BRCA2* genes to low doses of ionizing radiation during mammography and the consequent development of a breast tumor. This study included 213 women previously diagnosed with causal variants of the *BRCA1* and *BRCA2* genes, who began screening with mammograms between 25 and 30 years. Of these, 85 had been diagnosed with breast cancer at least a year ago. All carriers, at the beginning of the study, provided information on the number of mammograms performed so far, the initial age at which they had their first mammogram and the number performed in the previous 12 months. For those who had been diagnosed with breast cancer less than a year before, exposure was calculated by subtracting from the total number of mammograms they had undergone in their lifetime, the number of them received in the year prior to the cancer diagnosis of the breast. The authors obtained a relative risk of 0.94, finding no association regarding cancer development and exposure to the mammography dose [42].

Another retrospective cohort study conducted by John et al. in 2013 investigated the influence of chest radiographs on the development of breast cancer. In this study, 454 carriers of mutations in the *BRCA1* gene and 273 carriers of mutations in the *BRCA2* gene were included under 50 years of age, and 261 had a personal history of breast cancer. Again, the authors found no relationship between having chest radiographs and the development of breast cancer before the age of 50 years [43].

Additionally, a study carried out by Giannakeas et al., (2014) investigated whether exposure of women diagnosed with mutations in the *BRCA1* and *BRCA2* genes to examinations with ionizing radiation, such as mammography, increase the risk of breast cancer. This study included 2346 women, 1844 of whom had a mutation in the *BRCA1* gene and 502 in the *BRCA2* gene, all without breast cancer. Their story of mammography exposure was analyzed, and all were followed to observe the diagnosis of cancer. The results showed that there is no association between prior exposure to mammography doses and the risk of breast cancer for *BRCA1* (hazard ratio of 0.79) and *BRCA2* carriers (hazard ratio of 0.9). Additionally, it was observed that having a mammogram at an earlier age, before 30 years old, does not increase the risk of developing cancer for carriers of mutations in both genes [44].

In contrast to the results previously presented, with no association between exposure to ionizing radiation and the development of cancer, the retrospective cohort study carried out by Andrieu et al., (2006) included 1601 carriers (1187 *BRCA1* mutation carriers and 414 *BRCA2* mutation carriers) and analyzed the relationship between having chest radiographs, excluding mammography, and an increased risk of developing breast cancer. Of 1601 women, 853 had been diagnosed with breast cancer. The authors concluded that any exposure to chest X-ray radiation led to an increased likelihood of developing breast cancer relative to the general population, with a hazard ratio of 1.54. They also concluded that, in women who underwent this examination under the age of 20 years old, the risk of developing primary breast cancer further increased. Thus, this study supports the hypothesis that carriers of *BRCA* gene mutations are more radiosensitive than the general population [45].

Later, in 2011, a study carried out by Lecarpentier et al., studied the risk of developing breast cancer depending on several factors such as the position of the mutation in the *BRCA1* and *BRCA2* genes, alcohol, smoking, and previous exposure to examinations involving ionizing radiation, namely chest radiography. The variation in breast cancer risk based on the location of the mutation was evaluated considering the information present in the study GENEPSO. In the study, 990 carriers were included and analyzed, 379 affected by breast cancer and 611 unaffected. Considering the risk of cancer and the position of the mutation, for the *BRCA1* gene, there was a central region in which the risk was lower (codons 374–1161), while for the *BRCA2* gene, there was strong evidence for a region in which the risk decreased (codons 957–1827) and a region with increased risk (codons 2546–2968). In this way, the study showed that, in fact, the risk of breast cancer is lower in the central regions of the *BRCA1/2* genes. Additionally, exposure to chest x-rays radiation was found to increase the risk of breast cancer, with a hazard ratio of 4.29, as well as smoking, with a hazard ratio of 2.09. Thus, it can be said that lifestyle, such as tobacco use, as well as exposure to ionizing radiation affects the development of cancer in individuals with mutations in the *BRCA*1 and *BRCA2* genes [46].

Additionally, a more detailed study carried out by Pijpe et al., was the retrospective cohort study, the GENE-RAD-RISK Project, in which the authors studied 1993 female carriers of mutations in the *BRCA1* and *BRCA2* genes over 18 years old, who had already been included in three large studies–GENEPSO (Paoli-Calmettes Institute, Marseille, France), EMBRACE (Centre for Cancer Genetic Epidemiology, Cambridge, United Kingdom) and HEBON (Departments of Clinical Genetics/Family Tumors, Netherlands). The authors observed a dose–effect relationship between any radiation exposure from a diagnostic test before the age of 30 years and the risk of developing breast cancer (hazard ratio of 1.90). They concluded that the risk of developing breast cancer increases with the increase in the number of chest X-rays performed under the age of 20 and 30 years, as well as the number of mammograms performed before the age of 30 years. However, no relationship was seen between carrying out diagnostic tests between the ages of 30 and 39 and the development of breast cancer [47].

Finally, in 2016 and 2017, Baert et al., developed two important studies, in which they obtained results that supported all other studies that had observed statistically significant differences in the radiosensitivity of individuals carrying *BRCA* mutations when they were exposed to diagnostic or therapeutic ionizing radiation. The study carried out in 2016 aimed to observe whether carriers of *BRCA1* mutations (*n* = 18) had hypersensitivity to ionizing radiation compared to non-carriers (*n* = 18). The study started with the definition of a new radiosensitivity indicator, RIND, measured through the micronucleus assay and based on the measurement of DNA repair and cell cycle inhibition capacities after exposure to doses of 2 and 4 Gy. The results showed the existence of a significant difference between the RIND score obtained for carriers and non-carriers of mutations in the *BRCA1* gene: 72% of the mutation carriers showed a radiosensitive phenotype while 72% of the control group did not appear to be radiosensitive. Additionally, it was also found that the radiosensitive phenotype was similar for individuals belonging to the same family [48]. A year later, in 2017, to see if the same results were obtained for carriers of mutations in the *BRCA2* gene, a second study was carried out using the same methods. The study analyzed blood samples from 18 patients with mutations in the *BRCA2* gene and 17 patients without mutations in the *BRCA1* and *BRCA2* genes, irradiating the peripherical blood lymphocytes with γ-radiation at a dose of 2 Gy originating from a cobalt-60 source. Then, a micronucleus assay was performed to assess the radiosensitivity of individuals, concluding that carriers of mutations in the *BRCA2* gene were more radiosensitive than non-carriers. However, a study with a larger population of subjects carrying these mutations should be performed [49]. Thus, the results obtained in both studies indicated that carriers of mutations in the *BRCA1/2* genes had, in fact, greater radiosensitivity compared to non-carriers. These results indicate that greater attention should be paid when considering the application of ionizing radiation, for diagnosis or therapy, in carriers of mutations in the *BRCA* genes.

**Table 1:** Summary of studies carried out that evaluated the risks associated with exposure to ionizing radiation, associated with diagnostic exams, in carriers of mutations in the BRCA1 and BRCA2 genes.

Authors	Year	Sample Size (*n*)	Gene	Exposure Type	Outcome
Narold et al. [41]	2006	3200 (1600 carriers with breast cancer and 1600 healthy carriers)	*BRCA1* and *BRCA2*	Mammography	No association was found between having a mammogram and breast cancer risk
Goldfrank et al. [42]	2006	213 carriers	*BRCA1* and *BRCA2*	Mammography	No association was found between mammogram exposure and breast cancer risk
John et al. [43]	2013	727 (454 *BRCA1* and 273 *BRCA2* mutation carriers aged <50 years)	*BRCA1* and *BRCA2*	Chest x-rays	No association was found between diagnostic chest x-rays and breast cancer risk before age 50 years
Giannakeas et al. [44]	2014	2346 (1844 *BRCA1* mutation carriers and 502 *BRCA2* mutation carriers)	*BRCA1* and *BRCA2*	Mammography	No significant association was found between prior mammography exposure and breast cancer risk for *BRCA1* or *BRCA2* carriers
Andrieu et al. [45]	2006	1601 carriers (1187 *BRCA1* mutation carriers and 414 *BRCA2* mutation carriers)	*BRCA1* and *BRCA2*	Chest x-rays	A positive association was found between diagnostic chest x-rays and breast cancer risk. In addition, the risk was increased in women aged 40 years and younger
Lecarpentier et al. [46]	2011	990 (379 affected by breast cancer and 611 unaffected)	*BRCA1* and *BRCA2*	Chest x-rays	An association was found between exposure to chest x-rays and the risk of breast cancer. A positive association was found between smoking and cancer risk
Pijpe et al. [47]	2012	1993 carriers	*BRCA1* and *BRCA2*	<0.0020 Gy, ≥0.0020–0.0065 Gy, ≥0.0066–0.0173 Gy, and ≥0.0174 Gy	A positive association was found between diagnostic chest x-rays before the age of 30 and breast cancer risk
Baert et al. [48]	2016	36 (18 carriers of *BRCA1* mutations and 18 non-carriers)	*BRCA1*	2 and 4 Gy	Healthy individuals with a *BRCA1* mutation show a significantly increased radiosensitivity compared with healthy controls
Baert et al. [49]	2017	35 (18 carriers of mutations in *BRCA2* gene and 17 non-carriers)	*BRCA2*	2 Gy	An increased radiosensitivity was found in *BRCA2* mutation carriers compared to non-carriers

### 3.2. Therapeutic Doses

Although radiotherapeutic approaches constitute a crucial tool for the treatment of oncological cases, through lesions in the DNA of tumor cells, including DSBs, they can lead to the development of another malignant neoplasm, namely a second breast cancer, due to the high radiation dose associated with this therapeutic option. Thus, some studies have been developed to study this subject (Table 2).

When a primary breast cancer is discovered and it needs to be surgically removed, individuals with mutations in the *BRCA1/2* genes have two options: breast-conserving surgery (BCS) or radical mastectomy. Often, after surgical removal, the individual may develop a second breast cancer, such as contralateral breast cancer, since during adjuvant radiotherapy, the individual is exposed to radiation.

In 2007, a study carried out by Broeks et al., evaluated whether mutations in genes involved in DNA repair pathways, such as the *BRCA1*, *BRCA2*, *CHEK2*, and *ATM* genes, make women more susceptible to contralateral breast cancer induced by ionizing radiation associated with radiotherapy. The study included women who developed contralateral breast cancer after having a primary breast cancer diagnosed before the age of 50, and who were treated (*n* = 169) or not (*n* = 78) with radiotherapy as treatment for their first breast tumor. Among the individuals with a history of radiotherapy, 24.3% had a mutation in one of the genes mentioned and among the individuals not submitted to radiation for the first breast tumor, 12.8% had some mutation. The results showed that carriers of mutations in genes involved in DNA damage repair have an increased risk of developing contralateral breast cancer after radiotherapy [50].

Additionally, in 2010, a retrospective cohort study by Pierce et al., was developed with the aim of evaluating and comparing the risk of developing a second contralateral breast cancer associated with the two mentioned surgical options. So, 655 carriers of mutations in the *BRCA* genes diagnosed with primary breast cancer were included in the study, 302 of whom opted for BCS, while 353 underwent radical mastectomy. All were followed up for assessment of local, regional, or systemic recurrence. The results obtained showed that the risk of developing contralateral breast cancer was higher for those who had undergone BCS, however, when considering the relative risk of developing contralateral breast cancer in individuals undergoing adjuvant radiotherapy, the same was not statistically significant. Additionally, the survival rates for each surgical procedure were also compared, with 92.1% and 87.3% for BCS, considering a period of 10 and 15 years after the procedure, respectively, and 91.8% and 89.9% for mastectomy, considering the same periods. Thus, it is concluded that carriers of mutations in the *BRCA* genes, with primary breast cancer, have similar survival rates for both procedures; however, women undergoing BCS have an increased risk of a second neoplastic event in the breast, which is reduced when they undergo radiotherapy [51].

Later, in 2011, another retrospective cohort study by Metcalfe et al., aimed to estimate the risk of developing contralateral breast cancer after 810 carriers of mutations in the genes under study, diagnosed with primary breast cancer, underwent radiotherapy, following one of the two therapeutic options previously discussed. In this study, it was found that 149 women (18.4%) developed contralateral breast cancer and that the risk of development considering a period of 15 years after diagnosis was 36.1% for carriers of mutations in the *BRCA1* gene and 28.5% for women with *BRCA2* gene mutations. Furthermore, women under 50 years of age at the time of diagnosis of their primary breast cancer, 15 years after diagnosis, were significantly more likely to develop contralateral breast cancer (37.6%) compared to women over 50 years of age (16.8%), and women aged 50 years with two or more first-degree relatives with early-onset breast cancer were at an increased risk of developing contralateral breast cancer (50%) compared with women who did not (36%). Additionally, oophorectomy, an ovarian cancer treatment option, led to a reduced risk of contralateral breast cancer in young women with mutations in the genes under analysis. Thus, it is concluded that the risk of developing contralateral breast cancer decreases with the age of *BRCA* mutation carriers at the time of diagnosis and with the performance of oophorectomy, increasing, in turn, with the number of first-degree relatives with the same diagnosis [52].

In 2013, another retrospective cohort study, developed by Bernstein et al., based on the WECARE study database, also studied the risk of developing contralateral breast cancer after radiotherapy in *BRCA* mutation carriers compared to non-carriers. The study included 603 women with contralateral breast cancer and 1199 women with unilateral breast cancer. Each participant completed a questionnaire based on their treatment history and tumor characteristics. Among the 603 cases, 96 (15.9%) carriers of deleterious mutations in the genes under analysis were identified, and 62 among the 1199 (4.4%). In women treated with radiation, the mean dose received was 1.1 Gy. The results show that the risk of developing contralateral breast cancer in women with the *BRCA1/2* deleterious mutation was four times greater, however, carriers exposed to radiation underlying radiotherapy for primary breast cancer did not have a significantly higher relative risk of contralateral breast cancer compared to unexposed carriers. Thus, the present study was not able to clarify whether carriers of deleterious mutations in the *BRCA1/2* genes were more susceptible to the carcinogenic effects caused by exposure to ionizing radiation than those without these mutations [53].

Finally, more recently, a study developed by Schlosser et al., (2020) aimed to analyze *BRCA* mutation carriers undergoing radiotherapy for breast cancer to determine whether they have a higher risk of developing a second primary neoplasm. The study included the analysis of 230 carriers who, in turn, had undergone radiotherapy for at least 5 years after being diagnosed with breast cancer. The results reported only six cases of women who developed a second primary neoplasm after radiotherapy: one papillary thyroid carcinoma, one esophageal adenocarcinoma, one stomach adenocarcinoma, two non-Hodgkin’s lymphomas, and one non-small cell lung cancer, all of which developed several years after irradiation and little were associated with radiation exposure. Thus, the results show that women with the mutation treated with radiotherapy for breast cancer do not have a statistically significant risk of a second primary malignancy induced by ionizing radiation [54].

Thus, despite the growing interest in trying to understand whether, in fact, exposure to ionizing radiation from radiotherapy causes some kind of effect in carriers of mutations in the *BRCA1/2* genes, so far the evidence is still unspecific, so further studies are needed to clarify this subject.

## 4. Conclusions

Some of the studies carried out show that there is an association between the exposure of individuals with HBOC to diagnostic and therapeutic doses of ionizing radiation and the development of cancer. Additionally, some studies have shown that age and the number of diagnostic tests performed, as well as smoking, influence the increased risk of developing cancer. In fact, the younger the patient is and the greater the number of diagnostic imaging tests performed, the greater the risk of developing primary breast cancer (Andrieu et al.). Thus, it would be expected that, currently, there are already specific and defined guidelines for carriers of mutations in the *BRCA1* and *BRCA2* genes. Despite that it is increasingly believed that these individuals, in fact, have greater radiosensitivity when compared to the general population, there is not enough and concrete evidence to adjust the current guidelines. Although there are several studies that show that it is essential that these same changes are carried out, many studies were not able to demonstrate the existence of an association between exposure to ionizing radiation and an increased risk of developing cancer in carriers of mutations in the *BRCA* genes. That said, it is extremely urgent to carry out more specific studies so that clear and objective conclusions can be obtained on this subject.

## Figures and Tables

**Table 2:** Summary of studies carried out that evaluated the risks associated with exposure to ionizing radiation, associated with radiotherapy, in carriers of mutations in the *BRCA1* and *BRCA2* genes.

Authors	Year	Sample Size (*n*)	Gene	Dose	Outcome
Broeks et al. [50]	2007	247 (169 treated with radiotherapy and 78 not treated)	*BRCA1, BRCA2, CHEK and ATM*	30.5–76 Gy	The risk of developing contralateral breast cancer after radiotherapy was higher for individuals carrying mutations in genes involved in DNA damage repair pathways.
Pierce et al. [51]	2010	655 carriers	*BRCA1* and *BRCA2*	Not disclosed	The risk of developing contralateral breast cancer was higher for individuals undergoing BCS compared to individuals undergoing mastectomy. The risk in individuals undergoing adjuvant radiotherapy was not statistically significant.
Metcalfe et al. [52]	2011	810 carriers	*BRCA1* and *BRCA2*	Not disclosed	The risk of developing contralateral breast cancer decreased with age at diagnosis, increasing with the number of first-degree relatives with the same diagnosis.
Bernstein et al. [53]	2013	1802 (603 with contralateral breast cancer and 1199 with unilateral breast cancer)	*BRCA1* and *BRCA2*	1.1 Gy (range = 0.02–6.2 Gy)	The risk of developing contralateral breast cancer in carriers was four times greater, however, carriers undergoing radiation therapy for primary breast cancer did not have a significantly higher relative risk of contralateral breast cancer.
Schlosser et al. [54]	2020	230 carriers	*BRCA1* and *BRCA2*	50 Gy (25 fractions, 2Gy per fraction (fx)) or 42.4 Gy for patients treated after 2010 (16 fractions, 2.65 Gy/fx) or 50.4 Gy for reconstructed or augmented breasts (28 fractions, 1.8 Gy/fx) or 45 Gy in 1.8 Gy/fx	Women with the mutation undergoing radiation therapy for breast cancer did not have a statistically significant risk of a second primary malignancy induced by exposure to ionizing radiation.

## Data Availability

Not applicable.
